# Three-Character Training of Question-Asking (TCT-Q) for Children with High-Functioning Autism Spectrum Disorder: A Randomized Controlled Trial

**DOI:** 10.3390/bs15111489

**Published:** 2025-10-31

**Authors:** Wanxue Hu, Yijie Wang, Siyuan Zhang, Siying Yu, Xinying Li

**Affiliations:** 1 State Key Laboratory of Cognitive Science and Mental Health, Institute of Psychology, Chinese Academy of Sciences, Beijing 100101, China; huwx@psych.ac.cn (W.H.); wangyj@psych.ac.cn (Y.W.); zhangsiyuan@psych.ac.cn (S.Z.); yusy@psych.ac.cn (S.Y.); 2 Department of Psychology, University of Chinese Academy of Sciences, Beijing 100049, China

**Keywords:** high-functioning autism spectrum disorder, question-asking, three-character training, social skills

## Abstract

Question-asking is a key component of social communication, and interventions targeting this skill may be able to improve social functioning in children with high-functioning autism spectrum disorder (HFASD). This study introduced a novel intervention method called the three-character training of question-asking (TCT-Q), aimed at teaching children with HFASD how to appropriately use 11 questions in social interactions. The effectiveness of TCT-Q was tested through a randomized controlled trial. Thirty-seven children were assigned to TCT-Q group (*n* = 19) or treatment as usual (TAU) group (*n* = 18). Children and their caregivers received two 60 min sessions weekly. Outcome variables were measured before training (T1), after training (T2), and three months after training (T3). Results showed that the question-asking frequency in the TCT-Q group increased significantly after the intervention (*p*s < 0.001), and the increase was significantly greater than that in the TAU group (*η_p_*^2^ = 0.089–0.370). Although the TCT-Q group showed greater numerical improvements in social communication and autistic mannerisms (*p*s < 0.05), the group-by-time interaction did not reach statistical significance. In conclusion, TCT-Q is a promising method for enhancing question-asking behaviors and social skills in children with HFASD.

## 1. Introduction

Among individuals with autism spectrum disorder (ASD), those with average or above-average language and cognitive abilities relative to their peers of the same age are generally considered high-functioning (Diehl et al., 2013). According to the Centers for Disease Control and Prevention (2025), 36.1% of ASD individuals undergoing cognitive assessments meet criteria for high-functioning autism spectrum disorder (HFASD). Despite their cognitive strengths, individuals with HFASD also face significant social challenges, such as avoiding eye contact, difficulties in maintaining conversations, and limited social reciprocity (Dalton et al., 2005; Diehl et al., 2013; Matson & Sturmey, 2022). Targeted social skill training is therefore essential for helping this subgroup address their social difficulties.

Question-asking is a fundamental component of social communication. It has many functions, including initiating interactions, conveying interest in a social partner, expressing empathy, and facilitating the identification of shared interests (Detar & Vernon, 2020; Doggett et al., 2013). The development of question-asking skills is not only closely related to social interaction, but also plays a role in shaping language, pragmatics, and adaptive skills (Verschuur et al., 2017). Typically, question-asking skills are acquired early in preschool years. However, research has shown that children with ASD exhibit persistent delays and deficits in this domain. Specifically, most children with ASD experience difficulties in question-asking, such as asking few questions and asking questions only for instrumental purposes like requesting or protesting (Bozkus-Genc & Yucesoy-Ozkan, 2021; Koegel et al., 2014). These deficits also exist in children with HFASD and significantly hinder their ability to engage in meaningful social interactions, which may have long-term negative implications for their social and cognitive development.

Existing intervention methods, such as Pivotal Response Treatment and Covert Audio Coaching, have incorporated question-asking skills as one of their goals and have achieved satisfactory results (Popovic et al., 2020; Mason et al., 2020). However, these approaches are broadly applicable to children with ASD, and the questions being taught are relatively simple, which may not fully meet the social needs of individuals with HFASD. We believe that, in addition to simple questions (e.g., ‘What’s that?’), it is also important to teach HFASD individuals to use two other types of questions. One type relates to events (e.g., ‘What did someone do?’), which helps them obtain detailed information about an event. The other type pertains to social interaction (e.g., ‘Do you like…?’), which assists them in initiating or maintaining conversations and interactive social exchanges.

For the purpose of teaching children with HFASD to use these three types of questions appropriately, we proposed a novel intervention method named three-character training of question-asking (TCT-Q). It is inspired by two classical Chinese texts for early education, the ‘Three-Character Classic’ and the ‘Standards for Students’, which distill knowledge and behavioral standards that children should keep in mind into three-character phrases. This three-character format exhibits the following features: (1) simplicity (e.g., ‘Man on earth, good at birth’, matching ‘人之初，性本善’ in Chinese); (2) rhythmic; and (3) structured (Y. Zhang, 2020). These features closely align with the strong preference for rhythm and structure of children with HFASD (Ding et al., 2024). Therefore, we encapsulated the questioning skills we aim to teach into catchy, highly structured three-character phrases in order to reduce HFASD children’s cognitive burden and increase their interest in learning social skills.

In addition to teaching children with HFASD, we also explain to caregivers the question-asking associated with these three-character phrases and then instruct them on how to use these phrases effectively. Previous research has demonstrated that caregiver-mediated interventions not only facilitate the generalization of skills across diverse contexts but also improve parental self-efficacy while reducing stress levels (Bradshaw et al., 2022; Pacia et al., 2022; Stadnick et al., 2015). Hence, we expect to maximize the effectiveness of the intervention and help children ask questions appropriately in real-life situations by assisting caregivers in implementing the three-character prompts during natural settings.

Overall, we present a novel cognitive behavioral therapy (CBT) based, caregiver-involved, question-asking training for children with HFASD. We conducted a randomized controlled trial (RCT) to evaluate the effectiveness of TCT-Q compared to treatment as usual (TAU). The objectives of this study were (1) to evaluate the feasibility of the study procedures and TCT-Q intervention in terms of participant recruitment, data collection, intervention completion, intervention fidelity, and intervention credibility; (2) to examine the effects of TCT-Q relative to TAU on question-asking (primary outcomes), social skills, and caregivers’ parenting stress (secondary outcomes).

## 2. Materials and Methods

The overall design of the TCT-Q is based on the principles of CBT. At the cognitive level, we employed techniques such as video modeling and role-playing to help children understand that certain needs (e.g., acquiring new information or seeking help) can be met by asking appropriate questions. These new understandings were condensed into three-character phrases to facilitate memory retention for the children. At the behavioral level, we created multiple scenarios (e.g., imaginative play or real-life situations) to evoke genuine needs in a specific context. Once needs were successfully evoked, we taught questioning techniques and provided immediate reinforcement. In behavior training, teachers and caregivers used three-character phrases as prompts to encourage children to ask suitable questions.

### 2.1. Session Planning and Preparation of Training Materials

We determined a total of 11 themes across three major types (examples see Table 1), each corresponding to a specific question to be taught to children. For each theme, we developed a short mnemonic consisting of two three-character phrases. The content of each lesson is highly structured, with the sequence outlined in Table 2. Moreover, we produced a one-minute video, created an imaginative game, and three role-playing scenarios for each theme.

### 2.2. Participants

Participants were recruited from Beijing, China. Children who met the diagnostic criteria of HFASD were invited to participate. Inclusion criteria were as follows: (a) aged five to nine years; (b) met the diagnostic criteria for ASD on the Autism Diagnostic Observation Schedule, Second Edition (ADOS-2); and (c) demonstrated an intelligence quotient (IQ) score of 80 or above, measured by either the Chinese version of the Wechsler Preschool and Primary Scale of Intelligence-Fourth Edition (WPPSI-IV) or the Wechsler Intelligence Scale for Children-Fourth Edition (WISC-IV).

The required sample size was determined using G*Power 3.1.9.7 (Faul et al., 2007). A power analysis indicated that to achieve a medium effect size (partial *η*^2^ = 0.25), with an alpha of 0.05 and 90% power, a total of 36 participants (3 measurements per participant) were needed. The analysis was based on a repeated measures ANOVA model assessing within-between group interactions for 2 independent groups.

### 2.3. RCT

This study used an RCT design to compare the effects of TCT-Q and TAU. Participants were randomly assigned to either the TCT-Q or the TAU group. Outcome variables were measured at three time points: prior to training (T1), immediately after training (T2), and three months after training (T3). The trial was approved by the regional ethics committee of the Institute of Psychology, Chinese Academy of Sciences (Identifier H23064), and written informed consent was obtained from caregivers.

**Figure 1:**
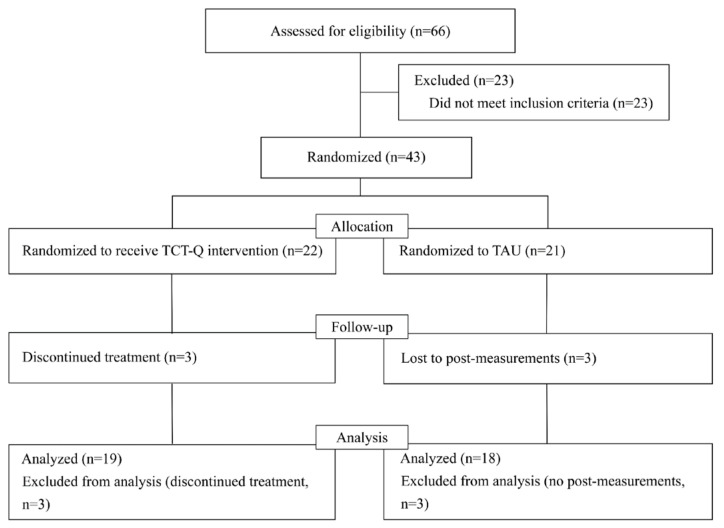
CONSORT diagram.

The procedure of the RCT is shown in Figure 1. For the TCT-Q group, intervention was conducted twice a week for 60 min per session, with each session focusing on one or two themes. The TAU group continued to receive their ongoing social-related interventions, such as PEAK, social story, and integrative intervention, attending 2.7 sessions per week on average. All participants in the TAU group were invited to receive social training after T3.

### 2.4. Measures

#### 2.4.1. Assessment Measures

1. Autism Diagnostic. ASD symptoms of the participants were evaluated using the ADOS-2, which is a standardized, semi-structured assessment tool (Lord et al., 2012). Only children with scores at or above the autism spectrum cut-off were eligible to participate in the study.
2. Intellectual ability. IQ was assessed using the Chinese version of WPPSI-IV or WISC-IV (Li & Zhu, 2014; H. Zhang, 2008). The population mean of IQ and index scores is 100, with a standard deviation of 15. The retest reliabilities of the Chinese version of the WPPSI-IV or WISC-IV (r = 0.76–0.91) and the inter-rater coefficients (0.96–0.99) are both satisfactory (Li & Zhu, 2014; H. Zhang, 2009).

#### 2.4.2. Feasibility Measures

Overall feasibility was calculated as (1) intervention completion; (2) dropout rates between the TCT-Q group and the TAU group post randomization; (3) intervention fidelity; (4) intervention credibility.

1. Fidelity of Implementation. Fidelity of Implementation (FoI) consists of 10 items, each scored as ‘0’ (fail) or ‘1’ (success). FoI assesses the complete implementation of the intervention process, correct feedback from the teacher on child/caregiver behavior, child’s cooperation with the session, and the child’s appropriate demonstration of skills in different contexts. A psychology graduate student assessed FoI on a random 25% of the TCT-Q session videos. An FoI score of ≥80% is considered acceptable (McGarry et al., 2020).
2. Intervention Credibility. The intervention credibility measure, designed by the researchers, consisted of a total of five questions. At T2 and T3, psychology graduate students conducted structured interviews with caregivers. The interview questions focused on the children’s question-asking and social behaviors in daily life, as well as caregivers’ feedback on the TCT-Q training.

#### 2.4.3. Outcome Measures

1. Primary outcome measures
  Number of question-asking. The numbers of children’s question-asking were assessed at all three time points (T1, T2, and T3). Each time, the teacher conducted a structured social interaction with the child, providing a total of 60 opportunities for the child to ask the target questions. Then, a caregiver was asked to interact with the child in a five-minute free-play activity as they would usually do, and was required to induce the child to ask as many target questions as possible they could. The interactions between the teacher/caregiver and child were videotaped, and the number of questions children asked was counted.
2. Secondary outcome measures
  (1) Social skills. The present study used the Chinese version of the Social Responsiveness Scale (SRS) for children (Cen et al., 2017). The SRS consists of 65 items, including five subscales (labeled social awareness, social cognition, social communication, social motivation, and autistic mannerisms), which are used to assess the social interaction ability of children with ASD (Constantino & Gruber, 2012). The lower the score, the less the degree of difficulty in social interaction for the individual. Caregivers completed the SRS at all three time points (T1, T2, and T3). The reliability of the total SRS score in the current study was 0.92.
  (2) Parenting stress. The Chinese version of Parenting Stress Index-Short Form (PSI-SF) (Ren, 1995), with a total of 36 items, was used to assess the levels of perceived parenting stress. The reliability of the total PSI-SF score in the current study was 0.93.

#### 2.4.4. Reliability Measures

Interobserver agreement. To assess interobserver agreement (IOA), two psychology graduate students counted the children’s question-asking numbers from video recordings independently. Twenty percent of videos were randomly selected for analysis. The researchers computed the IOA by dividing the number of agreements by the sum of agreements and disagreements and then multiplying the result by 100. An IOA ≥80% is considered acceptable (John et al., 2012).

### 2.5. Statistical Analyses

Data were analyzed using the statistical software SPSS version 22.0. Demographic variables were analyzed using independent *t*-tests for continuous variables and chi-square tests for categorical variables. Outcome variables were analyzed using repeated-measures analyses of variance (rmANOVA) with group (TCT-Q/TAU) as the between-subjects factor and time points (T1, T2, and T3) as the within-subjects factor.

## 3. Results

### 3.1. Participant Characteristics

A total of 37 participants completed the entire study, including 19 in the TCT-Q group and 18 in the TAU group. The overall mean IQ score was 107.54 (*SD* = 17.21), with a mean IQ of 107.84 (*SD* = 19.07) in the TCT-Q group and 107.22 (*SD* = 15.55) in the TAU group. There were no significant differences between the TCT-Q and TAU groups in gender, age, IQ score, or ADOS-2 score at T1 (Table 3).

### 3.2. Feasibility

Results showed good overall feasibility: a total of 66 children were enrolled in this study, and 28 were excluded for not meeting the inclusion criteria (*n* = 23) or for dropping out (*n* = 6). Of the 37 eligible children, 19 received TCT-Q and 18 received TAU (Figure 1). After randomization, the dropout rate was 14% in both the TCT-Q group and the TAU group. The FoI of the TCT-Q was 92%.

In addition, the intervention demonstrated good credibility. Caregivers’ feedback indicated notable improvements in children’s social behaviors following the intervention. First, caregivers observed a remarkable change in the overall number of questions posed by children. Second, children displayed greater politeness. They tend to seek others’ opinions before initiating social interactions, rather than directly starting social contact as they used to. Their social responses are also more contextually appropriate than before. Third, children exhibited heightened willingness to communicate with others, including caregivers, peers, and strangers. These positive changes were not only evident during the intervention period but were also sustained throughout the follow-up phase. Additionally, some caregivers attributed the improvement in their children’s social skills to the use of the three-character phrases.

### 3.3. Primary Outcome

The number of question-asking was assessed during teacher–child and caregiver–child interactions.

#### 3.3.1. Teacher–Child Interaction

As shown in Table 4, the results of the rmANOVA revealed a significant group-by-time interaction effect on the number of children’s question-asking, with a moderate effect size *(η_p_*^2^ = 0.370). Pairwise comparison analysis showed that at T1, there was no difference in the number of question-asking between the TCT-Q group and the TAU group (*p* = 0.773). At T2 and T3, the number of children who asked questions in the TCT-Q group was significantly greater than in the TAU group (*p_T_*_2_ = 0.001, *p_T_*_3_ = 0.029). These findings suggested that the TCT-Q intervention significantly increased the frequency of children’s question-asking during teacher–child interaction, with effects maintained after three months.

#### 3.3.2. Caregiver–Child Interaction

Similarly, the results of the rmANOVA (Table 4) revealed a significant group-by-time interaction effect on the number of children’s question-asking (*η_p_*^2^ = 0.089) during caregiver–child interaction. Pairwise comparison analysis showed that at T1, there was no difference in the number of question-asking between the TCT-Q group and the TAU group (*p* = 0.511). At T2, the TCT-Q group showed a significantly greater number of question-asking compared to the TAU group (*p* = 0.017). At T3, the TCT-Q group exhibited a marginally significant increase in question-asking compared to the TAU group (*p* = 0.056). The results indicated that the TCT-Q intervention notably enhanced the frequency of question-asking among children during interactions with their caregivers, with effects persisting after three months.

### 3.4. Secondary Outcome

#### 3.4.1. Social Skills

**Figure 2:**
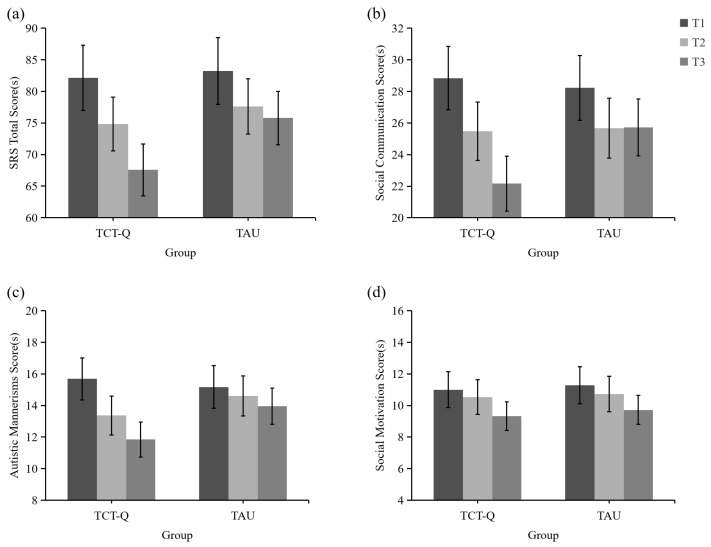
SRS scores of the two groups at three time points. (**a**) SRS Total scores; (**b**) Social Communication scores; (**c**) Autistic Mannerisms scores; (**d**) Social Motivation scores. TCT-Q: Three-Character Training of Question-Asking; TAU: Treatment As Usual. T1: before training, T2: after training, T3: three months after training.

Although there was a tendency for greater reduction in total SRS scores over time in the TCT-Q group compared to the TAU group (Table 4 and Figure 2a), the rmANOVA did not find any significant group difference. There was a significant main effect of time (*η_p_*^2^ = 0.215). Contrast analyses revealed a significant reduction from T1 to T3 (*p* = 0.001) and from T2 to T3 (*p* = 0.049) in the TCT-Q group, whereas no significant change was observed in the TAU group (*p_T_*_1*T*2_ = 0.412, *p_T_*_1*T*3_ = 0.189, *p_T_*_2*T*3_ = 0.903).

The sub-dimensions were further analyzed. A marginally significant group-by-time interaction effect was observed in the social communication scores (*η_p_*^2^ = 0.068). Contrast analyses showed a marginally significant decrease from T1 to T2 (*p* = 0.069), and a significant decrease from T1 to T3 and from T2 to T3 (*p_T_*_1*T*3_ < 0.001, *p_T_*_2*T*3_ = 0.022) in the TCT-Q group, while there was no difference in the TAU group (*p_T_*_1*T*2_ = 0.244, *p_T_*_1*T*3_ = 0.304, *p_T_*_2*T*3_ = 1.000). The results showed that the social communication scores for the TCT-Q group declined more than those for the TAU group over time (Table 4 and Figure 2b).

A significant main effect of time was found in the autistic mannerisms scores (*η_p_*^2^ = 0.150). Contrast analyses showed a marginally significant decrease from T1 to T2 (*p* = 0.073), and a significant decrease from T1 to T3 (*p* = 0.001) in the TCT-Q group, while there was no difference in the TAU group (*p_T_*_1*T*2_ = 0.930, *p_T_*_1*T*3_ = 0.565, *p_T_*_2*T*3_ = 0.899). Comparative analysis revealed a tendency of more reduction in autistic mannerism scores over time in the TCT-Q group compared to the TAU group (Table 4 and Figure 2c).

There was a significant main effect of time in the social motivation scores (*η_p_*^2^ = 0.116). Taking the two groups together, There was a significant reduction in the social motivation score, with an average decrease of 1.62 from T1 to T3 (*p* = 0.006, 95% CI = [0.499, 2.741]), followed by a 1.11 decrease from T2 to T3 (*p* = 0.028, 95% CI = [0.129, 2.081]), while no difference was observed from T1 to T2 (*p* = 0.397). The reduction in social motivation scores over time was similar in both the TCT-Q and TAU groups (Table 4 and Figure 2d).

For the social awareness scores, a marginally significant main effect of time was observed (*η_p_*^2^ = 0.098) in the whole sample (Table 4). Post hoc tests revealed a significant decrease of 0.95 on average from T1 to T3 (*p* = 0.061, 95% CI = [−0.044, 1.936]), but not from T1 to T2 (*p* = 0.109) or from T2 to T3 (*p* = 0.415).

There was no significant group-by-time interaction effect between the two groups in social cognition scores (*p* = 0.778) (Table 4). The time main effect and group main effect of social cognition scores were also not significant (*p_time_* = 0.103, *p_group_* = 0.321).

#### 3.4.2. Parenting Stress

While mean PSI-SF scores decreased over time in both groups, there were no significant group-by-time interaction effect (*p* = 0.560), time main effect (*p* = 0.117), or group main effect (*p* = 0.484) (Table 4). The results suggested that the TCT-Q did not increase parenting stress compared to other interventions.

### 3.5. Reliability

The IOA was 97% for teacher–child interactions and 86% for caregiver–child interactions.

## 4. Discussion

This study introduced a novel intervention method, named TCT-Q, aimed at enhancing question-asking in children with HFASD. Overall, the feasibility of the study was supported. Most participants completed the intervention, with dropout rates in both groups falling within an acceptable range. The intervention was implemented with adequate fidelity, and its credibility was considered satisfactory by participants. The RCT demonstrated a greater increase in question-asking frequency in the TCT-Q group compared to the TAU group. Some aspects of social skills also improved, although these improvements were not greater than those observed in the TAU group. These positive effects were maintained at the three-month follow-up, indicating a durable gain in question-asking and social skills. Furthermore, caregivers reported an improvement in children’s spontaneous and contextually appropriate questioning behaviors during daily interactions, suggesting the generalizability of the intervention effects.

### 4.1. Increased Question-Asking

To the best of our knowledge, most previous studies focusing on question-asking skills in ASD children with ASD did not distinguish between different ASD functioning levels (Kowitt et al., 2025; Pyles et al., 2021); thus, the TCT-Q is the first behavior training method specifically tailored to improve question-asking skills in children with HFASD. Among the three types of questions involved in TCT-Q, ‘simple questions’ have been the most commonly taught in existing methods (Popovic et al., 2020). Only a few methods teach ‘event-related questions’ (Bozkus-Genc & Yucesoy-Ozkan, 2021), and there are virtually no methods focusing on ‘social interaction questions.’ Because the latter two types of questions are important for children’s daily lives, including these questions in training may enhance the ecological validity of the intervention. Furthermore, while most prior studies employed a multiple-baseline design (Bross et al., 2022; Kowitt et al., 2025), the use of an RCT provides a more rigorous examination of the intervention’s effectiveness.

We believe that the main reason for TCT-Q’s effectiveness is its ability to leverage the cognitive strengths of children with HFASD. Utilizing children’s strengths for intervention is a concept that has gained popularity in recent years (Dawson et al., 2022; Patten, 2022). Two cognitive advantages of children with HFASD are directly related to TCT-Q. The first one is systemizing preference, which refers to their strong drive to analyze, understand, predict, control, and construct rule-based systems (Grove et al., 2018; Wheelwright et al., 2006). The skills involved in asking contextually appropriate questions are often ambiguous and flexible. Typically developing children acquire these skills naturally through social learning, whereas children with ASD usually find it challenging. The three-character phrases in the TCT-Q are highly structured and predictable, both in their form and semantic level (when a specific need arises, a certain question is asked). These features align well with the systemizing preference of children with HFASD (Strathearn et al., 2018), which can reduce the difficulty of learning question-asking and enhance their interest in learning. The second strength of children with HFASD that we utilized in TCT-Q is their rote memory. They are willing to memorize text materials with strong rhythm and cadence, just as the three-character phrases in TCT-Q. Therefore, they are more likely to keep the three-character phrases in mind and apply these questioning skills in daily life. A few other studies also developed social intervention methods that leverage the strengths of children with ASD, such as LEGO therapy and serious games, and have observed positive results. (Hu et al., 2018; Huo et al., 2021; Whyte et al., 2015). The success of these studies suggests that future intervention designs could benefit more from incorporating the strengths of children with ASD.

Another important factor contributing to the effectiveness of TCT-Q is the strong connection between the three-character phrases and Chinese culture. Unlike most existing interventions rooted in Western traditions, the three-character phrases used in TCT-Q draw inspiration from classical Chinese texts. These phrases are not only closely connected to children’s daily experiences but also carry a rhythmic cadence that makes learning more engaging and enjoyable (Y. Zhang, 2020). Such rhythm is particularly well-suited for high-functioning autistic children, as it facilitates both comprehension and memorization (Ding et al., 2024). In addition, many parents of autistic children were exposed to classical Chinese texts during their own schooling, which increases their receptivity to these three-character phrases and makes it easier for them to adopt and practice with their children in everyday life. By aligning with the Chinese cultural context, TCT-Q fosters active involvement from both children and parents, thereby enhancing its potential to serve and assist a wider population of families affected by autism in China.

### 4.2. Improvement of Social Skills

In addition to the proximal effect on question-asking behaviors, distal effects of TCT-Q on social skills, particularly social communication and autistic mannerisms, were also observed, although these effects did not reach statistical significance.

The improvement of social communication through question-asking behaviors may be mediated by enhanced conversational reciprocity. For example, when an HFASD child asks ‘What happened?’ during a conversation, it provides the other person with an opportunity to express themselves, thereby making the interaction more likely to become a bidirectional exchange. This speculation is supported by existing literature (Bozkus-Genc & Yucesoy-Ozkan, 2021; Detar & Vernon, 2020). In addition, questions like ‘Can I … (do something) with you?’ make children appear more polite and friendly, which can reduce social rejection from others and may contribute to improved social communication. Regarding the improvement in autistic mannerisms, we suspect that it is related to the use of questions like ‘Do you like …(something)?’ These questions make children inquire about others’ interests before initiating a conversation, thereby reducing rigid, stereotypical conversational behaviors (Gantman et al., 2012; Laugeson et al., 2012, 2015; Yamada et al., 2020; Yoo et al., 2014).

### 4.3. Caregiver Involvement

In recent years, many experts have advocated for caregivers to be the implementers of interventions (Bradshaw et al., 2022). In TCT-Q, we taught caregivers how to train their children in daily life, and we believe that the observed effects of TCT-Q are partly attributable to caregiver involvement. First, caregiver involvement increased the frequency of the intervention (Stadnick et al., 2015). Second, it provided more opportunities for children to generalize the question-asking skills they have learned in natural environments, both at home and outdoors. Third, for caregivers, the TCT-Q is not difficult to implement, so they are more willing to continue using it after the intervention, which may be an important reason for the observed long-term positive effects. Lastly, the skills caregivers learned from the TCT-Q influenced the way they communicate with their children. For example, they were more likely to induce their children to perform certain behaviors by creating opportunities and providing prompts, rather than asking them to do so via direct demands as before.

Although caregiver involvement is important, most intervention techniques seem challenging for them. In the current study, we trained caregivers to use three-character phrases as prompts in daily life to guide their children in asking appropriate questions. Since the three-character phrases are simple and easy to remember, this approach greatly reduces the difficulty for caregivers to implement the intervention.

### 4.4. Strengths and Weaknesses of the Study

The current study has several strengths. First, the intervention content is innovative, as it teaches children with HFASD to ask more complex and interactive questions. Second, it creatively leverages the cognitive strengths of children with HFASD, specifically systemizing preference and strong rote memory, by integrating the form of Chinese traditional textbooks with CBT in the course design. Third, training caregivers to serve as intervention implementers further enhances the overall effectiveness of the intervention. Lastly, the effectiveness of the intervention was rigorously tested through an RCT.

Despite these advantages, this study also has a few limitations. First, the structure and rhythm of the three-character phrases are closely related to the inherent characteristics of the Chinese language, and it remains uncertain whether they would retain their strong structural and rhythmic features when translated into other languages. Second, the overall sample size is relatively small; nevertheless, the results still demonstrate an effect on social skills. Third, most participants were from Beijing, and the number of female participants is limited, so the generalizability requires further validation. Lastly, the study’s follow-up lasted only three months, and the long-term effects of the intervention remain uncertain.

## 5. Conclusions

The TCT-Q intervention teaches children with HFASD to ask more complex and interactive questions by integrating the form of classic Chinese textbooks and CBT. Compared to some existing methods, TCT-Q is significantly more effective in teaching question-asking and has comparable effects in enhancing broader social skills. It is a promising new intervention method with positive application prospects.

## Figures and Tables

**Table 1:** TCT-Q theme examples.

Type	Theme	Mnemonic	Meaning
Simple	What’s this?	Ask the name, what is this 问名称，是什么	When we want to know the name of something, we could ask, “What’s this?”
Events- Related	What did … (someone) do?	What someone did, ask what 知某事，做什么	When we want to know what someone did, we could ask “What did … (someone) do?”
Social Interaction	Can I … (do something) with you?	Do it together, ask for the will 一起做，问意愿	When we want to do something with someone, we could ask “Can I … (do something) with you?”

**Table 2:** Procedure of a typical TCT-Q session.

Procedure	Description of the Procedure
Review	The teacher reviews the previous session’s content with the child.
Introduction	The teacher introduces a new question along with the corresponding three-character mnemonic.
Game	The teacher and assistant play imaginative games with the child using toys that the child likes. Obstacles are set up in the game where children are expected to say the target question to receive rewards.
Video Modeling	A one-minute video is shown to the child, illustrating the correct way to ask an appropriate question in a given situation. Then, the teacher explains the video and discusses it with the child.
Role-Playing	The child and the assistant engage in role-playing, including imitating the video setting and two other everyday situations, with the teacher providing guidance and feedback.
Caregiver Practice	Under the instruction of the teacher, the caregiver plays games and engages in role-playing with the child.
Homework	The teacher provides feedback on the caregiver’s homework from the previous session and assigns new homework.

**Table 3:** Demographic information of participants.

	TCT-Q (*n* = 19)	TAU (*n* = 18)	*t* or *χ*^2^	*p*
Age (years)	6.58 ± 1.35	6.17 ± 1.25	0.964	0.341
Gender, male	18	16	0.424	0.515
WISC-IV/ WPPSI-IV	107.84 ± 19.07	107.22 ± 15.55	0.108	0.915
ADOS-2	13.00 ± 4.97	12.89 ± 5.17	0.067	0.947

WISC-IV: Wechsler Intelligence Scale for Children—Fourth Edition; WPPSI-IV: Wechsler Preschool and Primary Scale of Intelligence—Fourth Edition; ADOS-2: Autism Diagnostic Observation Schedule—Second Edition; TCT-Q: Three-Character Training of Question-Asking; TAU: Treatment As Usual.

**Table 4:** Analysis of variance in the number of children asking questions, SRS, and PSI-SF scores between the two groups.

Outcomes	TCT-Q			TAU			Time *F*	Group *F*	Time × Group *F*
	T1 *M (SD)*	T2 *M (SD)*	T3 *M (SD)*	T1 *M (SD)*	T2 *M (SD)*	T3 *M (SD)*			
Number of question-asking (T-C)	17.42 (8.03)	30.47 (8.43)	27.74 (6.67)	18.22 (8.77)	18.39 (11.19)	21.39 (10.03)	29.31 ***	4.74 *	20.56 ***
Number of question-asking (C-C)	2.05 (2.42)	5.21 (4.17)	3.68 (2.58)	1.61 (1.50)	2.28 (2.76)	2.06 (2.41)	8.07 **	5.28 *	3.42 *
SRS Total	82.16 (24.31)	74.84 (20.55)	67.58 (21.22)	83.22 (20.31)	77.61 (15.93)	75.78 (13.66)	9.57 ***	0.48	1.09
Social Awareness	11.42 (3.73)	10.79 (2.35)	10.47 (2.76)	11.89 (2.30)	11.11 (1.91)	10.94 (2.62)	2.86 ^†^	0.32	0.02
Social Cognition	15.21 (5.91)	14.68 (3.99)	13.79 (4.85)	16.67 (4.27)	15.50 (3.85)	15.44 (3.73)	2.35	1.01	0.25
Social Communication	28.84 (10.35)	25.47 (9.18)	22.16 (8.95)	28.22 (6.55)	25.67 (6.68)	25.72 (5.90)	11.22 ***	0.19	2.55 ^†^
Social Motivation	11.00 (4.75)	10.53 (5.17)	9.32 (4.79)	11.28 (5.20)	10.72 (4.32)	9.72 (2.76)	4.59 *	0.05	0.02
Autistic Mannerisms	15.68 (5.14)	13.37 (5.23)	11.84 (5.26)	15.17 (6.36)	14.61 (5.52)	13.94 (4.41)	6.19 **	0.37	1.71
PSI-SF	93.58 (21.92)	93.26 (21.34)	91.90 (20.29)	91.28 (19.94)	89.06 (18.02)	85.39 (16.34)	2.29	0.50	0.59

T-C: Teacher–child; C-C: Caregiver–child; SRS: Social Response Scale; PSI-SF: Parenting Stress Index-Short Form; TCT-Q: Three-Character Training of Question-Asking; TAU: Treatment As Usual. * *p* < 0.05, ** *p* < 0.01, *** *p* < 0.001, ^†^ *p* < 0.09.

## Data Availability

The data presented in this study are available on request from the corresponding author (the data are not publicly available due to privacy or ethical restrictions).

## Abbreviations

The following abbreviations are used in this manuscript:

TCT-Q	Three-character training in question-asking
HFASD	High-functioning autism spectrum disorder
TAU	Treatment as usual
ASD	Autism spectrum disorder
CBT	Cognitive behavioral therapy
RCT	Randomized controlled trial
ADOS-2	Autism diagnostic observation schedule, second edition
IQ	Intelligence quotient
WPPSI-IV	Wechsler Preschool and Primary Scale of Intelligence-Fourth Edition
WISC-IV	Wechsler Intelligence Scale for Children-Fourth Edition
FoI	Fidelity of Implementation
SRS	Social responsiveness scale
PSI-SF	Parenting Stress Index-Short Form
IOA	Interobserver agreement
T-C	Teacher–child
C-C	Caregiver–child

## References

[B1-behavsci-15-01489] Bozkus-Genc G., Yucesoy-Ozkan S. (2021). The efficacy of pivotal response treatment in teaching question-asking initiations to young Turkish children with Autism Spectrum Disorder. Journal of Autism and Developmental Disorders.

[B2-behavsci-15-01489] Bradshaw J., Wolfe K., Hock R., Scopano L. (2022). Advances in supporting parents in interventions for autism spectrum disorder. Pediatric Clinics of North America.

[B3-behavsci-15-01489] Bross L. A., Huffman J. M., Anderson A., Alhibs M., Rousey J. G., Pinczynski M. (2022). Technology-based self-monitoring and visual supports to teach question asking skills to young adults with autism in community settings. Journal of Special Education Technology.

[B4-behavsci-15-01489] Cen C. Q., Liang Y. Y., Chen Q. R., Chen K. Y., Deng H. Z., Chen B. Y., Zou X. B. (2017). Investigating the validation of the Chinese Mandarin version of the Social Responsiveness Scale in a Mainland China child population. BMC Psychiatry.

[B5-behavsci-15-01489] Centers for Disease Control and Prevention (2025). Prevalence and early identification of autism spectrum disorder among children aged 4 and 8 years—Autism and developmental disabilities monitoring network, 16 sites, United States, 2022.

[B6-behavsci-15-01489] Constantino J. N., Gruber C. P. (2012). Social responsiveness scale (SRS) manual.

[B7-behavsci-15-01489] Dalton K. M., Nacewicz B. M., Johnstone T., Schaefer H. S., Gernsbacher M. A., Goldsmith H. H., Alexander A. L., Davidson R. J. (2005). Gaze fixation and the neural circuitry of face processing in autism. Nature Neuroscience.

[B8-behavsci-15-01489] Dawson G., Franz L., Brandsen S. (2022). At a crossroads—Reconsidering the goals of autism early behavioral intervention from a neurodiversity perspective. JAMA Pediatrics.

[B9-behavsci-15-01489] Detar W. J., Vernon T. W. (2020). Targeting question-asking initiations in college students with ASD using a video-feedback intervention. Focus on Autism and Other Developmental Disabilities.

[B10-behavsci-15-01489] Diehl J., Tang K., Thomas B. (2013). Encyclopedia of autism spectrum disorders.

[B11-behavsci-15-01489] Ding X., Wu J., Li D., Liu Z. (2024). The benefit of rhythm-based interventions for individuals with autism spectrum disorder: A systematic review and meta-analysis with random controlled trials. Frontiers in Psychiatry.

[B12-behavsci-15-01489] Doggett R. A., Krasno A. M., Koegel L. K., Koegel R. L. (2013). Acquisition of multiple questions in the context of social conversation in children with autism. Journal of Autism and Developmental Disorders.

[B13-behavsci-15-01489] Faul F., Erdfelder E., Lang A.-G., Buchner A. (2007). G*Power 3: A flexible statistical power analysis program for the social, behavioral, and biomedical sciences. Behavior Research Methods.

[B14-behavsci-15-01489] Gantman A., Kapp S. K., Orenski K., Laugeson E. A. (2012). Social skills training for young adults with high-functioning autism spectrum disorders: A randomized controlled pilot study. Journal of Autism and Developmental Disorders.

[B15-behavsci-15-01489] Grove R., Hoekstra R. A., Wierda M., Begeer S. (2018). Special interests and subjective wellbeing in autistic adults. Autism Research.

[B16-behavsci-15-01489] Hu X., Zheng Q., Lee G. T. (2018). Using peer-mediated LEGO^®^ play intervention to improve social interactions for Chinese children with autism in an inclusive setting. Journal of Autism and Developmental Disorders.

[B17-behavsci-15-01489] Huo C., Li Z., Meng J. (2021). Empathy interventions for individuals with autism spectrum disorders: Giving full play to strengths or making up for weaknesses?. Advances in Psychological Science.

[B18-behavsci-15-01489] John O. C., Heron T. E., Heward W. L. (2012). Applied behavior analysis second edition.

[B19-behavsci-15-01489] Koegel L. K., Park M. N., Koegel R. L. (2014). Using self-management to improve the reciprocal social conversation of children with autism spectrum disorder. Journal of Autism and Developmental Disorders.

[B20-behavsci-15-01489] Kowitt J. S., Madaus J., Simonsen B., Freeman J., Lombardi A., Ventola P. (2025). Implementing pivotal response treatment to teach question asking to high school students with Autism Spectrum Disorder. Journal of Autism and Developmental Disorders.

[B21-behavsci-15-01489] Laugeson E. A., Frankel F., Gantman A., Dillon A. R., Mogil C. (2012). Evidence-based social skills training for adolescents with autism spectrum disorders: The UCLA PEERS^®^ program. Journal of Autism and Developmental Disorders.

[B22-behavsci-15-01489] Laugeson E. A., Gantman A., Kapp S. K., Orenski K., Ellingsen R. (2015). A randomized controlled trial to improve social skills in young adults with autism spectrum disorder: The UCLA PEERS^®^ program. Journal of Autism and Developmental Disorders.

[B23-behavsci-15-01489] Li Y., Zhu J. (2014). The Chinese version of the Wechsler preschool and primary scale of intelligence-4th edition.

[B24-behavsci-15-01489] Lord C., Rutter M., DiLavore P. C., Risi S., Gotham K., Bishop S. L. (2012). Autism diagnostic observation schedule, second edition (ADOS-2) manual: Modules 1–4.

[B25-behavsci-15-01489] Mason R. A., Gregori E., Wills H. P., Kamps D., Huffman J. (2020). Covert audio coaching to increase question asking by female college students with autism: Proof of concept. Journal of Developmental and Physical Disabilities.

[B26-behavsci-15-01489] Matson J. L., Sturmey P. (2022). Handbook of autism and pervasive developmental disorder.

[B27-behavsci-15-01489] McGarry E., Vernon T., Baktha A. (2020). Brief report: A pilot online pivotal response treatment training program for parents of toddlers with Autism Spectrum Disorder. Journal of Autism and Developmental Disorders.

[B28-behavsci-15-01489] Pacia C., Holloway J., Gunning C., Lee H. (2022). A systematic review of family-mediated social communication interventions for young children with autism. Review Journal of Autism and Developmental Disorders.

[B29-behavsci-15-01489] Patten K. K. (2022). Finding our strengths: Recognizing professional bias and interrogating systems. The American Journal of Occupational Therapy.

[B30-behavsci-15-01489] Popovic S. C., Starr E. M., Koegel L. K. (2020). Teaching initiated question asking to children with autism spectrum disorder through a short-term parent-mediated program. Journal of Autism and Developmental Disorders.

[B31-behavsci-15-01489] Pyles M. L., Chastain A. N., Miguel C. F. (2021). Teaching children with autism to mand for information using “why?” as a function of denied access. The Analysis of Verbal Behavior.

[B32-behavsci-15-01489] Ren W. (1995). Parenting stress, coping strategy and satisfaction of parent-child relations. Unpublished Master Dissertation.

[B33-behavsci-15-01489] Stadnick N. A., Stahmer A., Brookman-Frazee L. (2015). Preliminary effectiveness of Project ImPACT: A parent-mediated intervention for children with autism spectrum disorder delivered in a community program. Journal of Autism and Developmental Disorders.

[B34-behavsci-15-01489] Strathearn L., Kim S., Bastian D. A., Jung J., Iyengar U., Martinez S., Goin-Kochel R. P., Fonagy P. (2018). Visual systemizing preference in children with autism: A randomized controlled trial of intranasal oxytocin. Development and Psychopathology.

[B35-behavsci-15-01489] Verschuur R., Huskens B., Verhoeven L., Didden R. (2017). Increasing opportunities for question-asking in school-aged children with autism spectrum disorder: Effectiveness of staff training in pivotal response treatment. Journal of Autism and Developmental Disorders.

[B36-behavsci-15-01489] Wheelwright S., Baron-Cohen S., Goldenfeld N., Delaney J., Fine D., Smith R., Weil L., Wakabayashi A. (2006). Predicting autism spectrum quotient (AQ) from the systemizing quotient-revised (SQ-R) and empathy quotient (EQ). Brain Research.

[B37-behavsci-15-01489] Whyte E. M., Smyth J. M., Scherf K. S. (2015). Designing serious game interventions for individuals with autism. Journal of Autism and Developmental Disorders.

[B38-behavsci-15-01489] Yamada T., Miura Y., Oi M., Akatsuka N., Laugeson E. A. (2020). Examining the treatment efficacy of peers in Japan: Improving social skills among adolescents with autism spectrum disorder. Journal of Autism and Developmental Disorders.

[B39-behavsci-15-01489] Yoo H. J., Bahn G., Cho I. H., Kim E. K., Kim J. H., Min J. W., Lee W. H., Seo J. S., Jun S. S., Bong G., Cho S., Shin M. S., Kim B. N., Kim J. W., Park S., Laugeson E. A. (2014). A randomized controlled trial of the Korean version of the PEERS^®^ parent-assisted social skills training program for adolescents with ASD. Autism Research.

[B40-behavsci-15-01489] Zhang H. (2008). The Chinese version of the Wechsler intelligence scale for children—4th edition.

[B41-behavsci-15-01489] Zhang H. (2009). The revision of WISC-IV Chinese version. Psychological Science.

[B42-behavsci-15-01489] Zhang Y. (2020). Teaching research and case design of the three-character classic from the perspective of international communication. Doctoral Dissertation.

